# Total energy expenditure of 10- to 12-year-old Japanese children measured using the doubly labeled water method

**DOI:** 10.1186/s12986-017-0226-y

**Published:** 2017-11-15

**Authors:** Keisuke Komura, Satoshi Nakae, Kazufumi Hirakawa, Naoyuki Ebine, Kazuhiro Suzuki, Haruo Ozawa, Yosuke Yamada, Misaka Kimura, Kojiro Ishii

**Affiliations:** 1 Graduate School of Health and Sports Science, Doshisha University, Kyoto, Japan; 2 Department of Early Childhood Education, Kyoto Bunkyo Junior College, Kyoto, Japan; 3 Department of Nutrition and Metabolism, National Institutes of Biomedical Innovation, Health and Nutrition, Tokyo, Japan; 4 Faculty of Health and Medical Sciences, Kyoto Gakuen University, Kyoto, Japan; 5 Faculty of Health and Sports Science, Doshisha University, Kyoto, Japan; 6 Faculty of Education, Art and Science, Yamagata University, Yamagata, Japan; 7 School of Management, Shizuoka Sangyo University, Shizuoka, Japan

**Keywords:** Total energy expenditure, Doubly labeled water, Fat-free mass, Fat mass, Deuterium, Pre-adolescent children, Estimated energy requirement, Physical activity level

## Abstract

**Background:**

To establish Japanese children’s estimated energy requirements, total energy expenditure (TEE) data measured using the doubly labeled water (DLW) method is needed. This study aimed to 1) obtain basic TEE data from Japanese children measured using DLW (TEE_DLW_), 2) compare TEE_DLW_ with TEE estimated by various estimation formulas to calculate their accuracy, and 3) develop a new equation to estimate TEE using body composition and pedometers.

**Methods:**

TEE was measured using DLW in 56 10- to 12-year-old Japanese children (33 boys, 23 girls). Physical activity level (PAL) was calculated by dividing TEE_DLW_ by estimated resting energy expenditure. To assess their physical activity, participants wore pedometers during the 7-d DLW period. Total body water was calculated from ^2^H and ^18^O; fat-free mass (FFM) and fat mass (FM) were then determined.

**Results:**

In boys and girls of normal weight, TEE_DLW_ was 2067 ± 230 kcal/d and 1830 ± 262 kcal/d, respectively. Average PAL was 1.58 ± 0.17. FFM was strongly related to TEE (*r* = 0.702, *p* < 0.01). After adjusting for FFM and FM, step count was significantly associated with TEE (*r* = 0.707, *p* < 0.01). The TEE estimation formula used in the Dietary Reference Intakes (DRI) for the United States and Canada estimated TEE_DLW_ with high accuracy (bias: 2.0%) in both sexes. We developed new equations for TEE consisting of FFM and step count, which accounted for 68% and 65% of TEE variance in boys and girls, respectively: boys, 47.1 × FFM (kg) + 0.0568 × step count (steps/d) – 122, and girls, 55.5 × FFM (kg) + 0.0315 × step count (steps/d) - 117.

**Conclusions:**

The TEE in 10- to 12-year-old Japanese children measured using DLW was approximately 7% lower for boys and 12% lower for girls compared to the current Japanese DRI. If PAL can be accurately determined, the equation in the DRI for the United States and Canada may be applicable to Japanese children. In addition, TEE could be predicted using FFM and step count.

## Background

Estimated energy requirements (EER) as indicated in Dietary Reference Intakes for Japanese (Japan-DRI) [1] are defined as “habitual energy intake in a day which is predicted to have the highest probability that energy balance (energy intake − energy expenditure, in adults) becomes zero in a group [2].” EER can be estimated from dietary assessment by assuming that the energy intake and energy requirement are equal when weight does not fluctuate substantially over a short time, but this method underestimates EER [3]. Therefore, energy intake is assumed to equal total energy expenditure (TEE), and generally EER is estimated from TEE [4]. In children, when estimating EER from TEE, energy deposition for growth must be added (EER = TEE + energy deposition) [5].

Doubly labeled water (DLW) is the most accurate TEE estimation method under free-living conditions [6, 7], but it is expensive and requires specialized analysis equipment [8], making large-scale data collection difficult. The Japan-DRI refers to only 2 reports of DLW data from Japanese children [9, 10]. Consequently, the EER of Japanese children is determined based on other nationalities. To establish the EER of Japanese children, data using the DLW method must be collected to serve as a gold-standard population reference.

In the current Japan-DRI, children’s TEE is estimated by multiplying basal metabolic rate (BMR), determined by multiplying the sex- and age-specific BMR standard value per unit body weight by body weight, by the physical activity coefficients (PA) determined by the physical activity level (PAL) [4]. In obese adults, estimation using the BMR standard was reported to overestimate BMR [11]. However, the accuracy of the BMR standard to estimate TEE in children remains unknown. Moreover, the possible appropriateness of other TEE estimation formulae [5, 12] for children in Japan has not been established [4].

The largest component of TEE is typically BMR, which is determined by body size and composition, particularly fat-free mass (FFM) [13], and the inter-individual variability of TEE adjusted using FFM (or BMR) indicates inter-individual differences in physical activity energy expenditure. Therefore, by measuring body composition and physical activity, TEE might be predictable to some extent without the DLW method.

The current study aimed to 1) obtain baseline TEE data from Japanese children with the DLW method, 2) examine the accuracy of previously proposed TEE estimation equations, and 3) develop a new TEE estimation equation for 10- to 12-year-old children in Japan using body composition and pedometer data.

## Methods

### Participants

Physical activity levels significantly differ between rural and urban Japanese children [14]. Therefore, we recruited 62 healthy elementary school attendees (5th to 6th grade; age 10 to 12 years) in a rural area (Chiba prefecture) and an urban area (Hyogo prefecture). Fifth graders (*n* = 38) were measured in November 2006 (rural area, *n* = 36) and November to December 2007 (urban area, *n* = 2), and 6th graders (urban area, *n* = 24) were measured in February 2009, all during school days in a typical week. The inclusion criteria were healthy subjects without illness, with informed consent to participate in the study obtained from children and their parents. The experimental protocol compliant with the Code of Ethics of the World Medical Association (Declaration of Helsinki) and conducted with approval of the ethics committee of the Graduate School of Education, Hokkaido University (H18–04).

To assess their physical activity, participants wore pedometers (Omron, Kyoto, Japan) [15, 16] on their waist during the 7-d DLW period except when taking a bath or shower, swimming, and sleeping. We excluded subjects who spilled DLW during administration (*n* = 1), with stable isotope concentrations higher at 24 h than at 4 h after DLW administration (*n* = 3), who were absent on urine sample collection days (*n* = 2), or who wore the pedometer less than 3 days for ≥10 h per day during the 7-d DLW measurement period. The final dataset was obtained from 56 children (33 boys and 23 girls) for DLW and body composition data and from 52 children (31 boys and 21 girls) for step counts per day.

### Total energy expenditure measurements using DLW

Total energy expenditure was measured over 7 d as described previously [17, 18]. Height and body weight (BW) were measured in underwear on the day of DLW administration. Subjects were administered ~0.18 g/kg BW ^2^H_2_O (99.8 atom%; Taiyo Nippon Sanso, Tokyo, Japan) and ~3.6 g/kg BW H_2_^18^O (10.0 atom%; Taiyo Nippon Sanso). To ensure that all DLW was consumed, after the subject drank the DLW, we rinsed the container with a total of 50 mL commercial mineral water, which the subject also drank, and then repeated this procedure. Urine samples were collected before and 4 h, 1 d, 4 d, and 7 d after DLW administration. All participants included in the final dataset (*n* = 56) provided all five urine samples under a researcher’s or teacher’s supervision.

The urine samples were analyzed in duplicate or triplicate using stable isotope ratio mass spectrometry (Hydra 20–20, SerCon Ltd., Crewe, UK) with gas (H_2_ or CO_2_) equilibration methods, a platinum catalyst for H_2_, and commercially available stable isotope standards (Iso-Analytical, Crewe, UK). The average standard deviations (SD) were 0.25 ppm for ^2^H and 0.40 ppm for ^18^O. The ^2^H and ^18^O dilution spaces (Nd and No) were determined using the plateau method. The mean ± SD Nd/No in the present study was 1.031 ± 0.008 (range, 1.004–1.059), which is acceptable based on previous studies [19, 20]. Thus, total body water (TBW) (g) was calculated as the average of the value obtained by dividing the dilution space of ^2^H by 1.041 and the value obtained by dividing the dilution space of ^18^O by 1.007. TBW (mol) was obtained as TBW (g)/18.02, and carbon dioxide production rate (rCO_2_) (mol d^−1^) was calculated as 0.4554 × TBW (mol) × (1.007 × ^18^O elimination rate [ko] - 1.041 × ^2^H elimination rate [kd]), assuming that isotope fractionation applies only to breath water using eq. A6 by Schoeller et al. [21] with the revised dilution space constant provided by Racette et al. [19]. The average determinant coefficients (R^2^) of ko and kd were 0.997 and 0.995, respectively. The rCO_2_ (L d^−1^) was obtained as 22.4 × rCO_2_ (mol d^−1^). We assumed that the respiratory quotient (RQ) was 0.85 [22], and TEE was calculated using the modified Weir’s equation [23] as follows: TEE (kcal/d) = 1.1 rCO_2_ + 3.9 rCO_2_/RQ. The detailed quality checklist is described in International Atomic Energy Agency (IAEA) documents [24]. FFM was calculated using TBW with the age-dependent hydration factor of children [25]. Fat mass (FM) and percent fat (% fat) were calculated using FFM and BW. PAL was obtained by dividing TEE measured with the DLW method (TEE_DLW_) by resting energy expenditure (REE) from an estimation formula [26] obtained from Japanese children: for boys, 14.4 × BW (kg) + 5.09 × height (cm) – 34.0 × age (y) + 403, and for girls, 7.64 × BW (kg) + 4.22 × height (cm) – 22.5 × age (y) + 526. We assumed an age of 10 years for 5th graders and 11 years for 6th graders.

### Predictive equations of total energy expenditure

The applicability of the three predictive estimations of TEE is shown in Table 1.

**Table 1:** Predictive equations of total energy expenditure (TEE)

	Predictive equations
TEE_J-DRI_ (kcal/d) [4]	BMR standard^a^ (kcal/kg/d) × body weight (kg) × PA_J-DRI_^b^
TEE_IOM_ (kcal/d) [5]	For boys aged 9–18 y: 88.5–61.9 × age^c^ (y) + PA_IOM_^d^ × [26.7 × body weight (kg) + 903 × height (m)]
	For girls aged 9–18 y: 135.3–30.8 × age^c^ (y) + PA_IOM_^d^ × [10.0 × body weight (kg) + 934 × height (m)]
TEE_FAO_ (kcal/d) [12]	For boys aged 1–18 y: 310.2 + 63.3 × body weight (kg) - 0.263 × body weight (kg)^2^
	For girls aged 1–18 y: 263.4 + 65.3 × body weight (kg) - 0.454 × body weight (kg)^2^

^a^BMR standard is 37.4 kcal/kg/d for boys and 34.8 kcal/kg/d for girls [4]

^b^Physical activity coefficients (PA) determined by PAL in Japan-DRI (PA_J-DRI_) [4] are as follows: if PAL <1.55, PA = 1.45 (level I); if 1.55 ≤ PAL <1.75, PA = 1.65 (level II); and if 1.75 ≤ PAL, and PA = 1.85 (level III) for both boys and girls

^c^We assumed an age of 10 years for 5th graders and 11 years for 6th graders

^d^PA used in the DRI for the United States and Canada developed by the Institute of Medicine (IOM) (PA_IOM_) [5] are as follows: boys, sedentary (1.0 ≤ PAL <1.4, PA = 1.00), low activity (1.4 ≤ PAL <1.6, PA = 1.13), active (1.6 ≤ PAL <1.9, PA = 1.26), and very active (1.9 ≤ PAL <2.5, PA = 1.42); girls,: sedentary (PA = 1.00), low activity (PA = 1.16), active (PA = 1.31), and very active (PA = 1.56)

### Statistical analysis

Results are presented as means ± SD. Analysis of covariance (ANCOVA) was used to analyze sex differences adjusting for measurement timing, because the measurement sites (urban vs. rural) and seasons (Oct.-Nov. vs Feb.) were potential confounders. To examine factors related to TEE, we used partial correlation analysis, also adjusting for measurement timing. To standardize FFM, we treated FFM as a covariate, because the intercept of the linear regression of FFM (x) against TEE (y) significantly differed from zero [27]. To analyze the differences and relationships between TEE_DLW_ and each estimated TEE, repeated-measures analysis of covariance with Bonferroni correction and partial correlation coefficient, adjusting for measurement timing, was used. The accuracy of estimated TEE was evaluated using Bland-Altman plots and root mean squared error (RMSE) as follows: $RMSE=\sqrt{\varSigma {\left( predicted\  TEE- measured\  TEE\right)}^2/n\ }$. The relationship between BMI and bias (predicted TEE − measured TEE) was analyzed by partial correlation, adjusting for measurement timing. Multiple linear regression analyses for predicting TEE, FFM, and step counts were entered into the regression equation simultaneously. The threshold for statistical significance was *p* < 0.05. SPSS Statistics 23 software (IBM Inc., Japan, Tokyo) was used for statistical analysis.

## Results

Table 2 shows the physical characteristics, body composition, TEE, REE, PAL, and daily step counts. Subjects’ average height and body weight ranged from 100% to 106% of the corresponding reference values [4] of the Japan-DRI. Compared to the corresponding FFMs measured using bioelectrical impedance in a previous study [26], the present FFMs were slightly higher (112% for boys, 106% for girls). Step counts were similar to those of previously reported Japanese subjects (aged 8.9 ± 1.8, for boys: 12,152 ± 2804 steps/d, for girls: 10,408 ± 1808 steps/d) [28]. Six boys and one girl were overweight, and one boy was obese based on BMI cutoffs [29]. TEE_DLW_, predicted REE, and step counts were significantly higher in boys than girls; however, there was no significant sex difference for PAL (overall average PAL [*n* = 56], 1.58 ± 0.17). Excluding overweight and obese subjects, TEE_DLW_ was 2067 ± 230 kcal/d for boys and 1830 ± 262 kcal/d for girls.

**Table 2:** Characteristics of the subjects

	Boys		Girls		
	*n*	Mean ± SD	*n*	Mean ± SD	*p* ^*i*^
Height (cm)	33	142.6 ± 6.9	23	145.5± 6.6	0.265
Body weight (kg)	33	37.9 ± 6.7	23	36.7 ± 6.3	0.458
BMI^a^ (kg/m^2^)	33	18.6 ± 2.8	23	17.2 ± 1.9	0.092
Overweight [n (%)]		6 (18%)		1 (4%)	
Obesity [n (%)]		1 (3%)		0 (0%)	
FFM^b^ (kg)	33	31.9 ± 4.3	23	29.6 ± 4.0	0.110
FM^c^ (kg)	33	6.0 ± 3.7	23	7.1 ± 4.5	0.609
% fat (%)	33	15.1 ± 7.2	23	18.6 ± 9.4	0.387
TEE_DLW_-1^d^ (kcal/d)	33	2107 ± 273	23	1847 ± 269	0.002
REE^e^ (kcal/d)	33	1321 ± 113	23	1185 ± 69	0.000
PAL^f^	33	1.60 ± 0.16	23	1.56 ± 0.19	0.626
Step count (steps/d)^g^	31	12,823 ± 2945	21	10,526 ± 2493	0.009
TEE_DLW_-2^h^ (kcal/d)	26	2067 ± 230	22	1830 ± 262	0.004

^a^Subjects were classified based on BMI cutoffs [29]

^b^ *FFM* fat-free mass

^c^ *FM* fat mass

^d^Total energy expenditure measured by doubly labeled water of all subjects

^e^Resting energy expenditure was predicted by equation of Kaneko et al. [26]

^f^Physical activity level was calculated as TEE_DLW_ / predicted REE [26]

^g^We excluded the data of two boys and two girls because of insufficient pedometer wearing time

^h^TEE_DLW_ excluding overweight and obese subjects on the basis of BMI cutoffs [29]

^i^Analysis of covariance on each characteristics, adjusting for measurement timing

Table 3 shows the partial correlations between TEE and body size, body composition, and step count. After adjusting for measurement timing, FFM showed the highest correlation coefficient in both boys and girls. After adding FFM as a covariate, only step count was significantly associated with TEE for both sexes. This result did not change after adjustment with FM.

**Table 3:** Partial correlation between TEE (kcal/d) and height, body weight (BW), body composition and step count

Covariates	Subject	n	Height	BW	FFM^a^	FM^b^	% fat	Steps
none	Boys and girls	52	0.365^**^	0.521^**^	0.702^**^	0.088	−0.091	0.430^**^
	Boys	31	0.513^**^	0.609^**^	0.618^**^	0.379^*^	0.276	0.447^*^
	Girls	21	0.517^*^	0.425^*^	0.767^**^	−0.089	−0.314	0.129
MT^c^	Boys and girls	52	0.385^**^	0.535^***^	0.673^***^	0.178	0.006	0.388^**^
	Boys	31	0.356	0.619^***^	0.637^***^	0.381^*^	0.245	0.375^*^
	Girls	21	0.735^***^	0.675^**^	0.771^***^	0.311	0.038	0.075
MT^c^ and FFM	Boys and girls	52	−0.155	−0.065	–	−0.065	−0.054	0.695^***^
	Boys	31	−0.128	0.185	–	0.185	0.184	0.708^***^
	Girls	21	0.107	0.049	–	0.049	0.054	0.546^*^
MT^c^, FFM and FM	Boys and girls	52	−0.153	–	–	–	0.040	0.707^***^
	Boys	31	−0.123	–	–	–	0.012	0.696^***^
	Girls	21	0.097	–	–	–	0.023	0.548^*^

^a^Fat-free mass derived from total body water

^b^Fat mass calculated by subtracting FFM from body weight

^c^Measurement timing

^**^ *p* < 0.05, ^**^ *p* < 0.01, ^***^ *p* < 0.001

Table 4 shows the accuracy and association of each estimated TEE compared to TEE_DLW_. TEE_J-DRI_ and TEE_FAO_ significantly differed from TEE_DLW_. TEE_IOM_ demonstrated the smallest bias and RMSE (both sexes: bias, 2.0%; accurate estimation rate ≥ 90%). Partial correlation analysis indicated significant relationships between TEE_DLW_ and all estimated TEEs for both boys and girls.

**Table 4:** Differences and correlations between the predicted and measured total energy expenditure (TEE)

	TEE Mean (SD) kcal/d	Bias Mean [95% CI] %	RMSE^e^ kcal/d	Accurate estimation^f^ %	Under estimation^g^ %	Over estimation^h^ %	Correlation coefficient
Boys (*n* = 33)							
TEE_DLW_^a^	2107 (273)						
TEE predicted							
TEE_J-DRI_^b^	2264 (470)^*^	6.8 [2.6, 11.1]	302.8	63.6	3.0	33.3	0.885^†^
TEE_IOM_^c^	2153 (321)	2.0 [0.3, 3.7]	110.2	93.9	0.0	6.1	0.944^†^
TEE_FAO_^d^	2320 (279)^*^	10.9 [6.7, 15.0]	319.9	57.6	0.0	42.4	0.635^†^
Girls (*n* = 23)							
TEE_DLW_^a^	1847 (269)						
TEE predicted							
TEE_J-DRI_^b^	2007 (401)^*^	8.6 [2.9, 14.2]	297.8	69.6	4.3	26.1	0.854^†^
TEE_IOM_^c^	1882 (271)	2.0 [0.1, 3.9]	90.0	100.0	0.0	0.0	0.941^†^
TEE_FAO_^d^	2031 (183)^*^	11.5 [5.7, 17.4]	308.2	34.8	4.3	60.9	0.654^†^

^a^TEE measured by doubly labeled water (DLW) method

^b^TEE estimated by equation of Dietary Reference Intakes (DRI) for Japanese [4], basal metabolic rate (BMR) standard (kcal/kg/d) × body weight (kg) × PA_J-DRI_ (physical activity coefficient)

^c^TEE estimated by equation of Institute of Medicine (IOM) [5]

^d^TEE estimated by equation of FAO (Food and Agriculture Organization of the United Nations) [12]

^e^Root mean squared error

^f^Percentage of the subjects predicted by equation within ± 10% of measured TEE

^g^Percentage of the subjects predicted by equation < 90% of measured TEE

^h^Percentage of the subjects predicted by equation > 110% of measured TEE

^*^Significantly different from TEE_DLW_, *p* < 0.05 (repeated measures analysis of covariance with Bonferroni correction, adjusting for measurement timing)

^†^Signigicantly correlate with TEE_DLW_, *p* < 0.05 (Partial correlation coefficient, adjusting for measurement timing)

**Fig. 1:**
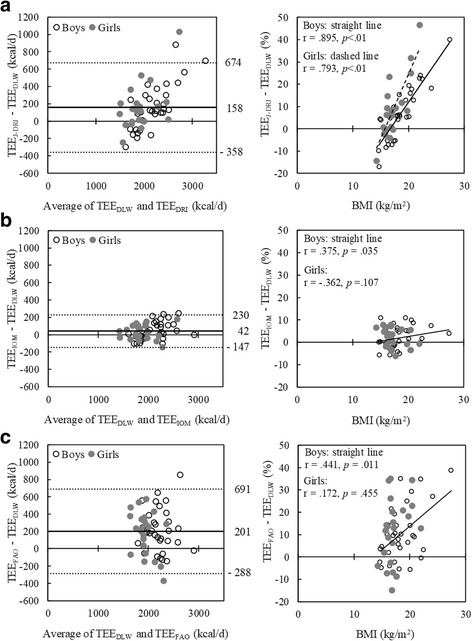
Bland-Altman plots and relationship between bias of total energy expenditure (TEE) and BMI. In the graph on the left, the thick straight line represents mean, and the dashed lines represent the lower and upper limits of agreement (± 2 standard deviations). **a** TEE estimated using the equation of Dietary Reference Intakes (DRI) for Japanese [4]. **b** TEE estimated using the Institute of Medicine (IOM) equation [5]. **c** TEE estimated using the Food and Agriculture Organization of the United Nation (FAO) equation [12]. In the graph on the right, the relationship between bias (predicted minus measured TEE) and BMI was examined using partial correlation analysis, adjusting for measurement timing

Fig. 1 shows Bland-Altman plots using three predictive equations and the relationships between BMI and bias (predicted TEE − measured TEE). The IOM equation had the smallest difference in the mean (42 kcal/d) and limits of agreements (−147 to 230 kcal/d). The range of limits of agreement was similar for the FAO equation (−288 to 691 kcal/d) and Japan-DRI (−358 to 674 kcal/d). Bias was strongly related to BMI in both sexes for TEE_J-DRI_, whereas this relationship was weakly significant in boys and not significant in girls for both TEE_IOM_ and TEE_FAO_. TEE_IOM_ estimated TEE within ± 10% bias even for overweight or obese individuals.

To predict TEE, FFM and step count were entered into the multiple regression analysis simultaneously (Table 5). For boys, the TEE (kcal/d) predictive equation was 47.1 × FFM (kg) + 0.0568 × step count (steps/d) - 122, and for girls, 55.5 × FFM (kg) + 0.0315 × step count (steps/d) – 117, which accounted for 68% and 65% of the TEE variance, respectively. Standard errors were 277 kcal/d for boys and 333 kcal/d for girls.

**Table 5:** Multiple linear regression analysis for predicting total energy expenditure (kcal/d) in 10- to 12-year-old children

	Boys and girls (*n* = 52)			Boys (*n* = 31)			Girls (*n* = 21)		
Predictor variables	B	β	*p*	B	β	*p*	B	β	*p*
FFM (kg)	51.1	0.74	0.000	47.1	0.73	0.000	55.5	0.83	0.000
Steps	0.0505	0.51	0.000	0.0568	0.61	0.000	0.0315	0.28	0.049
Constant	−177	–	0.000	−122	–	0.664	−117	–	0.729
	Adjusted R^2^ = 0.712			Adjusted R^2^ = 0.679			Adjusted R^2^ = 0.654		

*FFM* fat-free mass, *B* partial regression coefficient, *β* standardised partial regression coefficient

All predictor variables were entered into the regression equation simultaneously

## Discussion

We found that TEE_DLW_ of 10- to 12-year-old Japanese children was lower than current Japan-DRI criteria [4]. Furthermore, the IOM TEE equation [5] was applicable to Japanese children, and TEE could be predicted to some extent using FFM and step count.

The TEE indicated by the Japan-DRI is 2210 kcal/d for boys and 2070 kcal/d for girls [4]. Compared to the TEE_DLW_ of non-overweight or -obese children in the present study (boys: 2067 ± 230 kcal/d, girls: 1830 ± 262 kcal/d), TEE of the Japan-DRI was approximately 7% higher for boys and 12% higher for girls. Moreover, TEE_J-DRI_ overestimated TEE_DLW_ (Table 4). The only study of TEE in Japanese children aged 10 to 12 years cited by the Japan-DRI reported that TEE at an average age of 11.2 ± 1.0 years (boys: *n* = 5, girls: *n* = 7) was 1968 ± 299 kcal/d [9], which is lower than the TEE of the Japan-DRI, also suggesting that the EER of 10- to 12-year-old children in the current Japan-DRI may overestimate actual energy requirements.

FFM was reported to predict about 60% of TEE in elementary school children [27, 30]. While we also found a significant relationship between FFM and TEE, FFM explained 40–50% of inter-individual TEE variability in the present study, due to differences in participants’ range of FFM. FM also relates to TEE, because greater body size affects both REE and activity-related expenditure (AEE) through cost of weight-bearing activities [31]. These relationships are supported by the finding that children’s FFM was related to TEE, REE, and AEE, regardless of ethnicity [27]. Hence, the current and previous studies indicate that FFM is the major determinant of TEE in elementary school children.

We used step count as a physical activity index, as in previous studies [32, 33]. Step count was significantly related with TEE in both sexes after adjusting for FFM and FFM + FM, suggesting step count can explain inter-individual differences other than body size. Indeed, predictive equations consisting of FFM (kcal/d) and daily step count could account for 65% or more of TEE variance (Table 5). Previous studies reported that non-locomotive activity significantly impacted PAL [32], and girls’ step counts were not significantly related to PAL [33]. Thus, the predictive equation might be improved by adding measurements of non-locomotive activities, such as active standing or organized sports. Since approximately 90% of non-locomotive activities are light-intensity physical activity and strongly related with sedentary time [34], measurement of sedentary time may also be useful.

If PA can be accurately determined, the IOM equation estimates TEE with good accuracy and limited influence of BMI, but even after obtaining PAL from DLW measurements, the Japan-DRI equation overestimates approximately 30% of children because the BMR standard it employs is a multiple of the weight determined to fit the reference weight and has no intercept [4]. Therefore, as reported in a study of Japanese adults [35], individuals who deviate from the reference weight have greater error, with increasing TEE overestimation in the overweight and underestimation in the underweight.

While the FAO formula [12] similarly overestimates TEE of Japanese children by about 10%, it is advantageous in that it does not require PAL estimation.

The IOM estimation formula [5] of TEE for children aged 9 to 18 years is based on data measured with the DLW method in 525 American subjects in the 5th to 85th BMI percentile. Bandini et al. [36] reported that bias (TEE_IOM_ - TEE_DLW_) was −5.8 ± 7.9% and accurate estimation was 70% in 161 girls aged 8 to 12 years, when using DLW-derived PAL for calculation. The accuracy of this estimation method has not been previously evaluated in a Japanese population [4]. In the present study, the average bias between TEE_IOM_ and TEE_DLW_ was 2.0%, and the rate of accurate estimation exceeded 90% (Table 4). In addition, the IOM formula estimated TEE with an error within ± 10% and a small influence of subjects’ BMI (Fig. 1), suggesting that it is useful for estimating TEE in Japanese 10- to 12-year-old children.

There are several limitations to this study. First, because we did not obtain the participants’ birth dates, we assumed ages of 10 years for 5th grade and 11 years for 6th grade in the REE and IOM equations. In Japan, the 5th grade classes include 10- and 11-year-olds, and 6th grade classes include 11- and 12-year-old children. Assuming that all 5th graders were 11 and all 6th graders were 12 years old, the average REE value would be −34 kcal/d (−2.6% compared with present data) for boys and −23 kcal/d (−1.9%) for girls, the average TEE_IOM_ value would be −62 kcal/d (−2.9%) for boys and −31 kcal/d (−1.6%) for girls, and the average PAL value would be +0.04 (+2.7%) for boys and +0.03 (+1.9%) for girls. Second, in TEE estimation by the DLW method, RQ is often substituted with the food quotient obtained from meal records, while the present study applied a factor of 0.85. However, it has been reported that the estimation error in this case was slight [22]. Third, when estimating PAL, we used estimated REE instead of the measured BMR value. Although the estimation error is considered small [26], the presence of some error must be acknowledged. The current Japan-DRI cited only research that actually measured BMR, while the TEE estimation formula used in the DRI for the United States and Canada included data that estimated BMR [5]. Fourth, the target age was limited to 10 to 12 years. It is unknown whether the results of this study can be applied to other age groups. In the future, data should be collected from children of various ages.

## Conclusions

Our findings suggest that the IOM equation provides a more accurate estimation of TEE in Japanese 10-to 12-year-olds than the current Japan-DRI. We further derived a new TEE predictive equation based on FFM and step count per day for this population, the validity of which requires further investigation.

## Abbreviations

% fat: Percent fat
ANCOVA: Analysis of covariance
B: Partial regression coefficient
BMR: Basal metabolic rate
BW: Body weight
DLW: Doubly labeled water
DRI: Dietary Reference Intakes
EER: Estimated energy requirements
FAO: Food and Agriculture Organization of the United Nations
FFM: Fat-free mass
FM: Fat mass
kd: ^2^H elimination rate
ko: ^18^O elimination rate
Nd: ^2^H dilution space
No: ^18^O dilution space
PA: Physical activity coefficients
PAL: Physical activity level
rCO_2_: Carbon dioxide production rates
REE: Resting energy expenditure
RMSE: Root mean squared error
RQ: Respiratory quotient
TBW: Total body water
TEE: Total energy expenditure
TEE_DLW_: Total energy expenditure measured by doubly labeled water
TEE_FAO_: Total energy expenditure predicted by equation of Food and Agriculture Organization of the United Nations
TEE_IOM_: Total energy expenditure predicted by equation of Dietary Reference Intakes for the United States and Canada, Institute of Medicine
TEE_J-DRI_: Total energy expenditure predicted by equation of Japan Dietary Reference Intakes
β: Standardized partial regression coefficient
